# The Effect of Anterior Closing Wedge Slope-Reducing Osteotomy on Coronal Alignment—Effect of Osteotomy Technique, Starting Point, and Degree of Correction

**DOI:** 10.1177/23259671251358401

**Published:** 2025-07-29

**Authors:** James Matthew Helm, Mark Coggins, Brian Crowley, Maile Curbo, Jacob Siahaan, Alexis Aboulafia, Alfred A. Mansour

**Affiliations:** † University of Texas Health Science Center Houston, Houston, Texas, USA; Investigation performed at the University of Texas Health Science Center Houston, Houston, Texas, USA

**Keywords:** anterior closing wedge osteotomy, closing wedge 3-dimensional simulation model, slope-reducing osteotomy, tibial osteotomy

## Abstract

**Background:**

Techniques for anterior closing wedge slope-reducing osteotomy (ACW-SRO) remain variable regarding management of the tibial tubercle and osteotomy starting point. Moreover, the potential unintended effect on coronal alignment has not yet been determined.

**Purpose:**

To determine the effect of the ACW-SRO technique and starting point on coronal alignment in knees with an elevated posterior tibial slope (PTS).

**Study Design:**

Descriptive laboratory study.

**Methods:**

Full-length lower extremity computed tomography scans were retrospectively reviewed in patients presenting to our level 1 trauma center to identify patients with an elevated PTS of ≥12° without secondary trauma to the lower extremity. Materialise software was used to generate 3-dimensional models and simulate supratubercle, transtubercle, and infratubercle ACW-SROs. Six osteotomies per tibia were simulated, with 3 using an anterior start point centered at the tibial tubercle, and 3 using a start point at the perfect anterior-posterior mid-axis point of the tibia (-AP). The PTS was corrected to 6° universally. Coronal alignment was measured using the medial proximal tibial angle (MPTA) before and after osteotomy.

**Results:**

Eleven tibias were included, with a mean native PTS of 14.5° (range 12°-18°). Transtubercle-AP and infratubercle-AP osteotomies had the largest mean ΔMPTA of 1.72° of varus (range, 0°-3°; *P* = .03) and 1.82° of varus (range 0°-3.5°; *P* = .03), respectively. There was a strong positive correlation between the degree of PTS correction and ΔMPTA. Supratubercle-AP, transtubercle-AP, and infratubercle-AP had the strongest correlations (0.77, *P* = .005; 0.66, *P* = .03; 0.68, *P* = .02, respectively). The mean ΔMPTA increased varus in all 6 osteotomies in tibias with PTS corrections of ≥9°.

**Conclusion:**

Isolated ACW-SRO can affect coronal alignment of the knee by introducing additional varus, particularly in transtubercle and infratubercle osteotomies utilizing the AP starting point. This is especially apparent in tibias requiring larger PTS correction. The tibial tubercle-referenced starting point may minimize coronal changes.

**Clinical Relevance:**

This simulated study showed that coronal alignment is affected by the ACW-SRO technique and starting point in patients with elevated PTS. All osteotomies created additional varus, which must be considered when planning PTS correction.

The posterior tibial slope (PTS) refers to the anterior-posterior (AP) angulation of the tibial plateau with respect to the tibial shaft and is commonly measured on lateral radiographs or magnetic resonance imaging of the lower extremity.^4,7,9^ A radiographic PTS measurement of ≥12° is a well-documented risk factor for both primary anterior cruciate ligament (ACL) injury and ACL graft reconstruction failure.^11,12,22^ Anterior closing-wedge slope-reducing osteotomy (ACW-SRO) is a surgical procedure that can decrease the PTS and reduce the risk of ACL graft failure by decreasing anterior tibial translation and the stress forces seen by the ACL graft.^8,10,18^ A 2023 systematic review of 34 papers by Mandalia et al^12^ reported a normal PTS range of 6° to 12° and also proposed 12° as the cutoff at which adjunct slope-reducing osteotomy should be considered during revision ligament reconstruction.

The primary goal of the ACW-SRO is isolated correction of the sagittal plane deformity in patients with an increased PTS. Many procedural variants have been described, mainly about the cephalocaudal relationship of the osteotomy start site in relation to the tibial tubercle (TT) (supratubercle, transtubercle, and infratubercle).^6,15,16,21^ ACW-SRO with concomitant or staged anterior cruciate ligament reconstruction (ACLR) procedures have also been described with successful outcomes.^5^ Dejour et al^5^ reported 9 patients who underwent combined ACW-SRO and revision ACLR and demonstrated a 100% union rate of the osteotomy site and no ACL graft failures at 4 years postoperatively. Akoto et al^1^ detailed a 2-stage procedure in which an ACW-SRO was followed by staged revision ACR with lateral extra-articular tenodesis, demonstrating good to excellent postoperative functional scores and no evidence of graft failure at 30 months postoperatively.

While the effect of ACW-SRO on the sagittal plane is well described, its potential unintended effect on the coronal plane is relatively unknown and must be considered, given the 3-dimensional (3D) anatomy of the tibiofemoral surfaces and biomechanical forces of the knee joint itself. There is currently a paucity of data on the effect of ACW-SRO on coronal plane anatomy. In 2023, Mayer et al^13^ reported a significant decrease in the medial proximal tibial angle (MPTA) in patients who underwent infratubercle ACW-SRO, reporting decreased preoperative MPTA and increased osteotomy height as predictive factors of a smaller postoperative MPTA. To our knowledge, no studies have yet defined the relationship between the ACW-SRO technique and coronal postoperative alignment. This study seeks to determine the effect of ACW-SRO and its associated factors on coronal alignment by simulating multiple osteotomies on computer-modeled tibias. We hypothesized that the osteotomy technique and start site, as well as the degree of correction, would have a significant unintended effect on coronal alignment. The objective of this study was to (1) determine the effect of isolated ACW-SRO on coronal alignment of the knee and (2) examine the relationship between coronal alignment change and factors such as the degree of PTS correction, the osteotomy technique and start point, and the TT position as measured by the tibial tubercle-trochlear groove (TT-TG) distance.

## Methods

### Patient Selection

This study was reviewed and approved by the institutional review board (IRB). Full-length lower extremity computed tomography (CT) scans of patients presenting to a level 1 trauma center were retrospectively reviewed to identify patients with an elevated PTS. Patients were included if their PTS was ≥12° and if there was no evidence of ipsilateral lower extremity trauma. Patients with a PTS of <12° and those with evidence of ipsilateral lower extremity trauma were excluded. The PTS was determined using a simplified technique described by Meier et al^14^ in 2020. The original study considered the medial and lateral PTS independently. For our study, we derived 1 PTS. From the sagittal view, we first created a line along the mechanical axis of the tibia. Using the same sagittal view, we added an intersecting line that was tangential to the anterior and posterior rims of the proximal tibia. The PTS was calculated as the angle between the line tangential to the proximal tibia rims and a line perpendicular to the mechanical axis of the tibia.^14^

### Modeling Software

Materialise Mimics software was used to generate 3D models from the CT scans, and Materialise 3-Matic was used to simulate supratubercle, transtubercle, and infratubercle ACW-SROs on each tibia. Both software programs, used in conjunction, have demonstrated the ability to generate accurate osteotomies in composite bone models to be used for biomechanical studies, but they have not been validated using in vivo models.^23^

### Osteotomy Techniques

**Figure 1.**
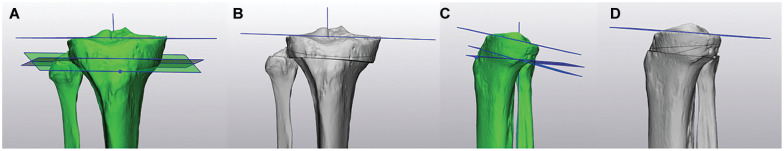
Supratubercle osteotomy simulated with the start point centered over the tibial tubercle. (A) Coronal view before osteotomy. (B) Coronal view after osteotomy. (C) Sagittal view before osteotomy. (D) Sagittal view after osteotomy. Cuts met at the posterior cortex of the tibia but did not go through the cortical flare.

**Figure 2. fig2-23259671251358401:**
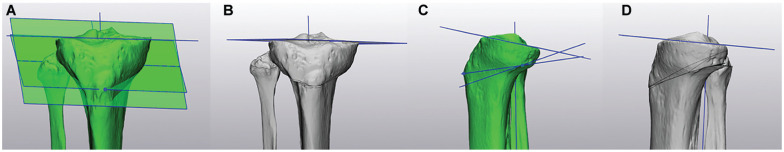
Transtubercle osteotomy simulated (A) coronal view before osteotomy, (B) coronal view after osteotomy, (C) sagittal view before osteotomy, and (D) sagittal view after osteotomy. Cuts met at the posterior cortex of the tibia but did not go through the cortical flare.

Each osteotomy was conducted with 2 different anterior starting points—a point centered over the TT (-tub) and a point centered over the perfect anterior-posterior mid-axis point (-AP) of the tibia; both of which result in a tubercle-sparing osteotomy. Simulated transtubercle osteotomies did not account for the AP plane change that would result from first performing a TT osteotomy (TTO). An ACW-SRO was simulated for each start point-tubercle relation combination for 6 osteotomies per tibia (Figures 1 [Fig fig2-23259671251358401]-3). The PTS was corrected to 6° universally.

**Figure 3.**
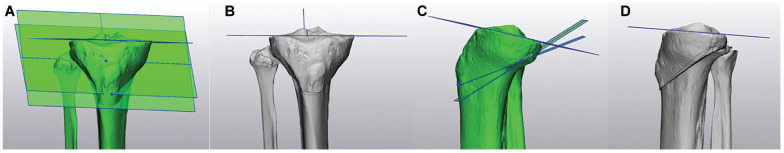
Infratubercle osteotomy. (A) Coronal view before osteotomy. (B) Coronal view after osteotomy. (C) Sagittal view before osteotomy. (D) Sagittal view after osteotomy. Cuts met at the posterior cortex of the tibia but did not go through the cortical flare.

### MPTA and TT-TG Measurement Techniques

Coronal alignment was measured using the MPTA before and after each osteotomy. To obtain the MPTA, we again used the technique described by Meier et al,^14^ by measuring the angle between the mechanical axis of the tibia and the articular surface of the proximal tibia in the coronal plane. The TT-TG distance and the degree of PTS correction were also measured and recorded for each tibia. The Sieberer et al^20^ 3D method for determining the TT-TG had not been published at the time our study was conducted; thus, the TT-TG was calculated using the traditional 2D method.^17^ The TG point was set at the deepest part of the TG on the axial slice with the most posterior medial and lateral femoral condyles. The TT point was set at the most anterior portion of the tibial tuberosity on any axial slice. The TT-TG distance was calculated by drawing 2 lines perpendicular to the intercondylar line, one to the TT point and the other to the TG point. The distance between the 2 perpendicular lines was used as the TT-TG value. Tibias were further subanalyzed for those with larger PTS corrections of ≥ 9° and TT-TG distances of ≥14 mm. Dandy reports 10 to 15 mm as a commonly accepted normal TT-TG range, and 14 mm was chosen because it falls within the normal range near but not at the upper limit of normal.^3^

### Statistical Analysis

Pearson correlation tests were utilized to determine the relationship between the PTS correction and MPTA change. The MPTA changes were analyzed with paired *t* tests and are based on the comparison of means with larger PTS corrections of ≥9° and TT-TG distances of ≥14 mm. The mean MPTA change that indicates significance is 1.7; therefore, this magnitude was the hypothesized significant difference between angles for tibias with a PTS correction of ≥9° and <9°. To achieve a statistical power of 80%, with significance at the 5% level, there would need to be at least 6 tibias per patient group with a total sample size of 12, accounting for a standard deviation of 1. All analyses were performed with an alpha level of .05, indicating statistical significance as <.05, in STATA 17.

## Results

Eleven tibias from 9 patients met the final inclusion criteria. Of the 11 tibias that met the inclusion criteria for the study, the mean preoperative PTS was 14.45° (range, 12°-18°), the mean TT-TG distance was 14.84 mm (range, 8.52-22 mm), and the mean preoperative MPTA was 88.36° (range, 87°-90°).

Transtubercle-AP and infratubercle-AP osteotomies introduced the greatest amount of MPTA change. Transtubercle-AP osteotomies introduced a mean of 1.73° of varus (range, 0°-3°; *P* = .03), and infratubercle-AP osteotomies introduced a mean of 1.82° of varus (range, 0°-3.5°; *P* = .03). All remaining osteotomy types resulted in overall mean varus change; nonetheless, none of these were statistically significant—supratubercle-AP, 1.36° (range, 0°-5°; *P* = .7); supratubercle-tub, 0.77° (range, 0°-2°; *P* = .5); transtubercle-tub, 1° (range, 0°-2.5°; *P* = .4); and infratubercle-tub, 1.27° (range, 0.5°-3°; *P* = .1) (Table 1).

**Table 1:** Osteotomy Type and the Mean Amount of MPTA Change*^*a*^*

ACW-SRO Type	Mean MPTA Change, Deg	*P*
Infratubercle, AP	1.82	**.03**
Transtubercle, AP	1.72	**.03**
Supratubercle, AP	1.36	.7
Infratubercle, tub	1.27	.1
Transtubercle, tub	1	.4
Supratubercle, tub	0.77	.5

a The bold *P* values indicate significance. ACW-SRO, anterior closing wedge slope-reducing osteotomy; AP, anterior-posterior; MPTA, medial proximal tibial angle; tub, tibial tubercle.

For all 3 osteotomy types, the MPTA change was greater when the start point was centered over the perfect AP mid-axis point of the tibia as opposed to the TT. However, when directly comparing the AP and tubercle starting points for each level of osteotomy, the MPTA change between them was not statistically significant at any level—infratubercle (AP: 1.81 ± 1.38 vs tub: 1.27 ± 0.96; *P* = .11); transtubercle, (AP: 1.73 ± 1.31 vs tub: 1 ± 0.63; *P* = .10), or supratubercle (AP: 1.36 ± 1.40 vs tub 0.77 ± 0.56; *P* = .08).

**Figure 4.**
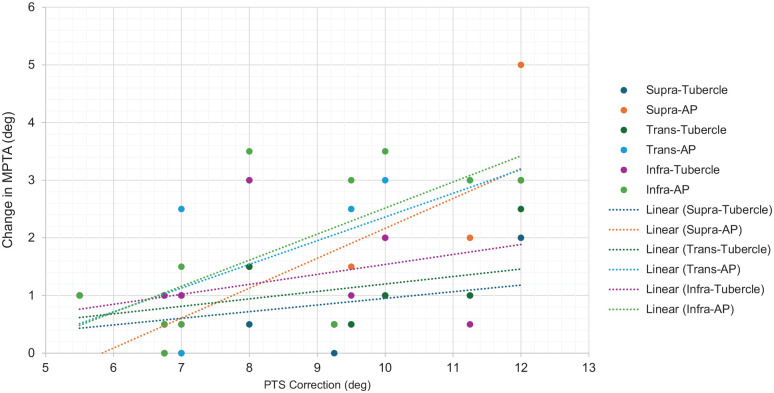
Correlation between PTS correction and change to the MPTA for supratubercle-AP, supratubercle-tub, transtubercle-AP, transtubercle-tub, infratubercle-AP, and infratubercle-tub ACW-SROs. ACW-SROs, anterior closing wedge slope-reducing osteotomies; AP, anterior-posterior; MPTA, medial proximal tibial angle; tub, tibial tubercle; PTS, posterior tibial slope.

There was a strong positive correlation between the degree of PTS correction and MPTA change, particularly for osteotomies with AP start sites. Supratubercle-AP, transtubercle-AP, and infratubercle-AP osteotomies had the strongest correlation coefficients of 0.77 (*P* = .005), 0.66 (*P* = .03), and 0.68 (*P* = .02), respectively. Comparatively, the correlation between the degree of PTS correction and MPTA change for tub start site osteotomies was much weaker. The correlation coefficients for supratubercle-tub, transtubercle-tub, and infratubercle-tub were 0.43 (*P* = .19), 0.43 (*P* = .19), and 0.38 (*P* = .25), respectively (Figure 4).

The amount of MPTA change introduced by each osteotomy type was further subanalyzed using 2 independent variables: (1) amount of PTS correction (≥9° vs <9°) and (2) native TT-TG distance (≥14 mm vs <14 mm). When tibias with a PTS correction of ≥9° were compared with tibias with a PTS correction of <9°, none of the 6 osteotomy types resulted in a statistically significant difference in the mean amount of MPTA change that was introduced: supratubercle-tub (PTS correction >9°: 0.90°± 0.74° vs PTS correction <9°: 0.83°± 0.52°; *P* = .86), supratubercle-AP (PTS correction ≥9°: 2.2°± 1.68° vs PTS correction <9°: 0.67°± 0.61°; *P* = .07), transtubercle-tub (PTS correction ≥9°: 1.1°± 0.82° vs PTS correction <9°: 0.92°± 0.49°; *P* = .66), transtubercle-AP (PTS correction ≥9°: 2.4°± 1.08° vs 1.17°± 1.29°; *P* = 0.12), infratubercle-tub (PTS correction >9°: 1.4°± 1.08° vs PTS correction <9°: 1.17°± 0.93°; *P* = 0.71), and infratubercle-AP (PTS correction >9°: 2.6°± 1.19° vs PTS correction <9°: 1.17°± 1.25°; *P* = .086) (Table 2). The mean change in MPTA between tibias with PTS changes of ≥9° versus <9° approached significance in the setting of supratubercle-AP and infratubercle-AP osteotomies simulations.

**Table 2:** Mean MPTA Change Introduced by Each Osteotomy Type in Tibias With a PTS Correction of ≥9°*^*a*^*

ACW-SRO Type	Mean MPTA Change in Tibias with PTS Correction ≥9, deg (n = 5)	Mean MPTA Change in Tibias With PTS Correction <9, deg (n = 6)	*P*
Infratubercle-AP	−2.6	−1.2	.08
Transtubercle-AP	−2.4	−1.2	.12
Supratubercle-AP	−2.2	−1.5	.06
Infratubercle-tub	−1.4	−1.2	.71
Transtubercle-tub	−1.1	−0.09	.66
Supratubercle-tub	−0.9	−0.8	.86

a ACW-SRO, anterior closing wedge slope-reducing osteotomy; AP, anterior-posterior; MPTA, medial proximal tibial angle; PTS, posterior tibial slope.

Similarly, none of the 6 studied osteotomies resulted in a statistically significant difference in the amount of MPTA change when tibias with a TT-TG of ≥14 mm were compared with tibias with a TT-TG of <14 mm: supratubercle-tub (TT-TG ≥14 mm: 1°± 0.61° vs TT-TG <14 mm: 0.75°± 0.61°; *P* = .52), supratubercle-AP (TT-TG ≥14 mm 1.5°± 1.89° vs TT-TG <14 mm: 0.92°± 0.74°; *P* = .27), transtubercle-tub (TT-TG ≥14 mm: 1°± 0.94° vs TT-TG <14 mm: 1°± 0.32°; *P*≥.999), transtubercle-AP (TT-TG ≥14 mm: 2.3°± 1.04° vs TT-TG <14 mm: 1.25°± 1.41°; *P* = .20), infratubercle-tub (TT-TG ≥14 mm: 1.2°± 1.04° vs TT-TG <14 mm: 1.33°± 0.98°; *P* = .83), and infratubercle-AP (TT-TG ≥14 mm: 2.2°± 1.15° vs TT-TG <14 mm: 1.33°± 1.37°; *P* = .29) (Table 3).

**Table 3:** Mean MPTA Change Introduced by Each Osteotomy Type in Tibias With a TT-TG of ≥14*^*a*^*

ACW-SRO Type	Mean MPTA Change in Tibias With a TT-TG of ≥14, deg (n = 5)	Mean MPTA Change in Tibias With a TT-TG of <14, deg (n = 6)	*P*
Infratubercle-AP	−2.2	−1.3	.83
Transtubercle-AP	−2.3	−1.25	.20
Supratubercle-AP	−1.5	−0.91	.29
Infratubercle-tub	−1.2	−1.3	.15
Transtubercle-tub	−1	−1	≥.999
Supratubercle-tub	−1	−0.75	.52

a ACW-SRO, anterior closing wedge slope-reducing osteotomy; AP, anterior-posterior; MPTA, medial proximal tibial angle; PTS, posterior tibial slope; TT-TG, tibial tubercle to trochlear groove; tub, tibial tubercle.

## Discussion

The most important finding was the unintended increase in varus that resulted from ACW-SRO. In particular, the osteotomies with the AP starting point produced more of a change in MPTA than the osteotomies with a tibial tubercle starting point.

Quinn et al^16^ compared supratubercle and transtubercle anterior closing wedge osteotomies and found that the 2 techniques were each associated with varying degrees of PTS correction when the same size osteotomy was performed, demonstrating that the technique and the start point must be considered and carefully scrutinized in these patients.^16^ In 2023, Mayer et al^13^ retrospectively reviewed 38 patients who underwent infratubercle ACW-SRO, reporting a mean decrease in MPTA of 1.3° after the procedure, showing that these osteotomies can indeed affect coronal alignment. They identified increased wedge size (degree of PTS correction) and lower initial MPTA (higher native varus) as predictive factors for increased amounts of postoperative MPTA change. Our study combines both aspects of these previous studies to advance the understanding of ACW-SRO and the specific surgical technique and preoperative factors that influence coronal change the most.

Although our study does not account for true patient factors such as soft tissue, body mass index, or fixation, the results of our study suggest all techniques produce a small degree of increased varus, with some specimens as high as 5°. Our study further suggests that transtubercle and infratubercle osteotomies may induce higher amounts of unintended coronal change than supratubercle osteotomies. This is likely because these osteotomies involve larger amounts of the proximal tibia and larger wedge sizes, providing more opportunity for the coronal plane to be impacted due to the changing 3D anatomy of the proximal tibia as the osteotomy is moved further distal. We take this a step further by not only comparing the type of osteotomy with relation to the TT, but also by the location of the initial starting point in the medial/lateral plane over the center of the TT or along the mid-axis of the tibia. Our results suggest that an osteotomy starting point centered over the TT rather than the mid-axis of the tibia may result in lower amounts of coronal change, especially when the degree of needed PTS correction is smaller. All osteotomies had higher amounts of MPTA change when their start point was centered over the mid-axis of the tibia. While these changes were not significant when specifically controlling for starting point, this may be a result of our small sample size. Further study is needed to validate this trend.

Additionally, there was a significant correlation between the degree of PTS correction and MPTA change. While this was weakly noted for all 3 osteotomies when the start point was centered over the TT (none of which reached statistical significance), it was significantly stronger for all osteotomies with the -AP start site (all 3 of which reached statistical significance), again suggesting that osteotomies with start points centered over the tibial tubercle may result in lower amounts of unintentional coronal change. This phenomenon is best demonstrated by the fact that the tibia we studied with the highest PTS (18°) had MPTA changes as large as 5° in some simulated osteotomies. Our data suggest that osteotomy technique and start point selection are even more critical in patients requiring larger PTS corrections. However, it is important to note that while supratubercle osteotomies were noted to cause the least amount of coronal change, a supratubercle osteotomy may not always be capable of providing an adequate amount of slope correction in patients with much higher PTS. In 2021, Quinn et al^16^ measured the wedge height required to induce 1° of change in the PTS, demonstrating that supratubercle osteotomies induced 1 degree of slope correction for every 1 mm of wedge cut, compared with the smaller 0.8 mm required to induce the same 1 degree of slope change with transtubercle osteotomies.^16^ In 2022, Weiler et al^24^ found no significant change to the MPTA after infratubercle and supratubercle ACW-SRO, but did report a statistically significant correlation between increased PTS correction and MPTA change. Furthermore, the Mayer et al^13^ study reports a correlation of 0.48 between wedge height and change in MPTA. Our findings were consistent with these proposed correlations regarding the amount of sagittal correction and degree of coronal change. However, while the previously published correlations established a correlative effect on coronal knee alignment relative to wedge size, our data begin to suggest an objective amount of PTS correction at which point one can start to expect a significant risk to coronal alignment. Further studies with larger study populations are needed to validate this.

When discussing osteotomy start points centered over the TT, it makes sense to also consider the anatomic variation of tubercle location in the medial/lateral plane from patient to patient. We attempted to further subanalyze our data with regard to the TT-TG distance to examine this. We found that the TT-TG distance did not have a statistically significant effect on the amount of MPTA change, regardless of the osteotomy performed. Previous studies have reported increased TT-TG values being associated with ACL graft failure.^2,19^ Chen et al^2^ reported a mean TT-TG of 11.2 mm in patients requiring ACL revision compared with 9.3 in the control population, demonstrating that increased TT-TG is independently associated with ACL graft failure. Our study suggests a positive correlation between TT-TG and coronal change, demonstrating that a TT-TG of ≥14 mm was associated with significant MPTA change after infra- or transtubercle-AP ACW-SROs. Again, additional higher-powered studies are necessary to validate this trend.

### Limitations

This preliminary study is largely limited by its use of imaging-based computer simulation. The advantage of this study design is that we were able to perform multiple osteotomies on a single specimen to minimize variability in outcome based on each tibia's morphology. Our study would be significantly strengthened by using CT scans obtained for preoperative planning in patients who undergo an actual osteotomy and comparing the actual MPTA change to the simulated change, but our group's practice does not include routine preoperative CT scans for surgical planning. Accordingly, a database was used to identify tibias that met the inclusion criteria and obtained a scan for indications unrelated to our study. Future studies should seek to continue to examine and validate these measurement trends in vivo surgeon-performed osteotomies that are subject to human error and anatomic bone and soft tissue forces. Standard measurement error is also a limitation of this study, especially when discussing value changes of single-digit degrees. Furthermore, due to this study’s simulation nature, we cannot determine the clinical significance of these values. Specifically for osteotomies simulated at the transtubercle level, the inability to simulate a TTO and adjust the AP dimension of the ACW-SRO accordingly limits the clinical utility of our results specific to that technique. Further research is necessary to determine whether or not these small changes in coronal alignment affect patient outcomes. Future studies might utilize larger sample sizes and/or compare simulated varus change to actual in vivo varus change using pre- and postoperative patient imaging. This information would improve the clinical relevance of our study's results and might allow clinicians to identify interventions that minimize unintentional change to the proximal tibia in the coronal plane.

## Conclusion

Our findings support the notion that ACW-SRO affects coronal alignment, specifically creating varus, and may be technique- and start-point-dependent. The data suggest that supratubercle osteotomies and osteotomies with start points centered over the tibial tubercle may impart the lowest degree of coronal change, especially when the degree of PTS correction is smaller. Overall, osteotomy technique, start point, and degree of required PTS correction must be considered and scrutinized when planning and evaluating patients for these procedures.
